# Yellow Fever and Cardiovascular Disease: An Intersection of Epidemics

**DOI:** 10.5935/abc.20180041

**Published:** 2018-03

**Authors:** Gláucia Maria Moraes de Oliveira, Roberto Muniz Ferreira

**Affiliations:** 1 Programa de Pós-Graduação em Medicina (Cardiologia) do Departamento de Clínica Médica da Faculdade de Medicina e do Instituto do Coração Édson Saad da Universidade Federal do Rio de Janeiro, Rio de Janeiro, RJ - Brazil; 2 Hospital Samaritano, Rio de Janeiro, RJ - Brazil

**Keywords:** Yellow Fever, Tropical Ecosystem, Arbovirus Infections, Aedes, Liver Failure, Kidney Failure, Chronic, Hemorrhage, Bradycardia, Drug-Eluting Stents / adverse effects

Arboviral diseases are an important public health problem, especially in tropical and subtropical countries, such as Brazil, where viruses of the family *Flaviviridae,* responsible for dengue fever, zika and yellow fever (YF), and of the family *Togaviridae*, which cause chikungunya, predominate. In recent years, the number of cases has increased because of several factors, of which environmental changes, such as deforestation and climate changes, disorderly occupation of cities with low hygiene-sanitary conditions, in addition to the increased mobility of international travelers, stand out. Such factors have allowed the colonization of new areas by vectors, mainly *Aedes aegypti*, which can be found in 80% of the Brazilian territory.^1,2^

Dengue virus, which has four different serotypes, has accounted for isolated epidemics or for co-infections in 1984-1985, 1997-1999 and 2004-2007. Chikungunya, whose virus originated in Africa, succeeded the dengue fever in Brazil in 2014, with similar clinical and laboratory presentation, hindering the differential diagnosis. In 2015, the first cases of zika were reported in Brazil. Table 1 summarizes the clinical manifestations of those arboviral diseases.^2-4^

**Table 1:** Clinical spectrum of dengue, chikungunya and zika

Arboviral disease	Clinical presentation	
	Mild forms	Severe forms
Dengue	High fever, myalgia, joint pain, nauseas, vomiting, skin rash, hemorrhagic manifestations, low platelet count	Organ failure (respiratory, heart, hepatic, hematologic, central nervous system), refractory shock and death
Chikungunya	The aforementioned manifestations + symmetrical pain in small and large joints, except for hemorrhagic syndrome	Nephritis, meningoencephalitis, Guillain-Barré syndrome and flaccid paralysis
Zika	Milder aforementioned manifestations, conjunctivitis	Neurological complications, such as microcephaly (newborn infants), Guillain-Barré syndrome, hearing loss

According to the World Health Organization, YF is endemic in Brazil since the year 1900, with sylvan and urban cycles, aggravated by the presence of *Aedes aegypti* in the cities. In the past decades, there has been a significant reduction in the number of cases because of the increase in vaccine coverage. However, the disease spread from endemic areas to the vicinities with similar ecological characteristics has enabled the emergence of the recent epidemic in the Brazilian states of Minas Gerais, Rio de Janeiro and São Paulo.^5^ Table 2 shows the signs and symptoms of YF, highlighting hepatic and renal failures, in addition to bleedings that occur in the more severe forms.

**Table 2:** Clinical spectrum of yellow fever and respective treatment site^14^

Form	Signs and symptoms	Laboratory changes	Treatment site
Mild / Moderate	Fever, headache, myalgia, nauseas, absent/mild jaundice	Low platelet count, moderate elevation of transaminases, normal or mildly elevated bilirubin levels	Outpatient clinic / hospital (ward)
Severe	All aforementioned, jaundice, severe hemorrhages, oliguria, reduced level of consciousness	Severe low platelet count, increased creatinine, significant elevation of transaminases	Hospital (ward / intensive care unit)
Malignant	All classic symptoms of the severe form intensified	All aforementioned, disseminated intravascular coagulation	Hospital (intensive care unit)

Most monkeys in Africa are resistant to the YF virus, differently from the neotropical species of primates of the Americas, which are more susceptible to fatal infections, mainly the *Alouatta* ssp, which serves as a sentinel species for the YF virus. In those animal models, YF is characterized by a hemorrhagic viral disease with multiple organ failure and cardiovascular shock, similarly to that affecting human beings. In *Rhesus* monkeys, marked lymphopenia has been reported preceding the spleen, liver, kidney and lymphoid tissue damages. Those findings are probably due to viral replication, release of cytokines, IL-4, IL-5, IL-6, IL-8, IL-12/23p40, IL-15, IL-17, G-CSF, GM-CSF, sCD40, RANTES, MCP-1 and INFƔ, and gene expression associated with immune response, ionic metabolism and apoptosis.^6,7^

Cardiovascular involvement in arboviral diseases was described in 1822 in YF, with myocardial impairment characterized by bradycardia. Later, Lloyd^8^ has reported prolongation of the atrioventricular conduction and ventricular repolarization changes. In 1965, bradycardia and hypotension were reported in chikungunya, and, in 1973, myocarditis, pericarditis and atrial fibrillation were reported in dengue fever.^9,10^ A recent systematic review has reported that cardiovascular manifestations are common in chikungunya, mainly hypotension, shock, arrhythmias, myocarditis, dilated cardiomyopathy and congestive heart failure with troponin level elevation.^11^ The histopathological assessment of the cardiac tissue of a fatal case of myocarditis and cardiogenic shock due to dengue fever in Brazil has shown muscular necrosis and interstitial edema with viral particles in cardiomyocytes and interstitial space, suggesting direct action of the virus in the myocardium.^12^ Cases of myocarditis, heart failure, arrhythmia, atrial fibrillation and ventricular and supraventricular tachycardia have been reported in zika.^13^

The varied clinical presentation of YF, from asymptomatic to severe forms, affects directly the disease’s therapeutic strategy. The malignant manifestations are associated with a mortality rate of up to 50%, requiring, thus, attention and differentiated care.^14^ Although the disease has no effective specific treatment, respiratory, hemodynamic, metabolic and hemostatic supports, in addition to appropriate control of comorbidities, are fundamental to establish the patient’s recovery. Moreover, the Ministry of Health criteria for outpatient clinic follow-up or hospitalization should be met in patients with heart diseases (Table 2).^14^ However, some particularities of clinical management do apply to those patients.

There is no study in the literature about the safest way to treat patients with coronary artery disease (CAD) and manifestations of YF. The experience in treating epidemics of other arboviral diseases in Brazil, however, could be a reference. In 2013, the Brazilian National Institute of Cardiology (Instituto Nacional de Cardiologia) issued recommendations for the use of antiplatelet drugs in patients with CAD and dengue fever, which were incorporated into the Ministry of Health Manual of Diagnosis and Clinical Management of dengue fever.^15^ In that document, the recommendations for suspension of antiplatelet drugs acknowledged the importance of different levels of platelet count essentially in patients with bare-metal or first-generation drug-eluting stents, who required at least 6 months of dual antiplatelet therapy to minimize the risk of thrombosis.^15^ Since then, the most frequent use of second-generation drug-eluting stents with everolimus or zotarolimus has allowed for shorter periods of dual antiplatelet therapy with the same safety level. Considering that low platelet count is one of the most important characteristics of all viral hemorrhagic fevers, those recommendations could also serve as a model for new recommendations for YF.^16^

Thus, the consideration of validated tools to assess the risks for hemorrhage and thrombosis after coronary stent implantation is a promising strategy. An example is the PRECISE-DAPT score, which uses hemoglobin, leukocyte count, age, creatinine clearance and history of bleeding as variables to estimate that risk. Scores < 25 are predictors of a low risk of bleeding and could identify patients who benefit from longer periods of dual antiplatelet therapy (6-12 months). However, scores ≥ 25 are associated with high rates of bleeding, indicating a shorter period of dual antiplatelet therapy (3-6 months).^17^

**Figure 1:**
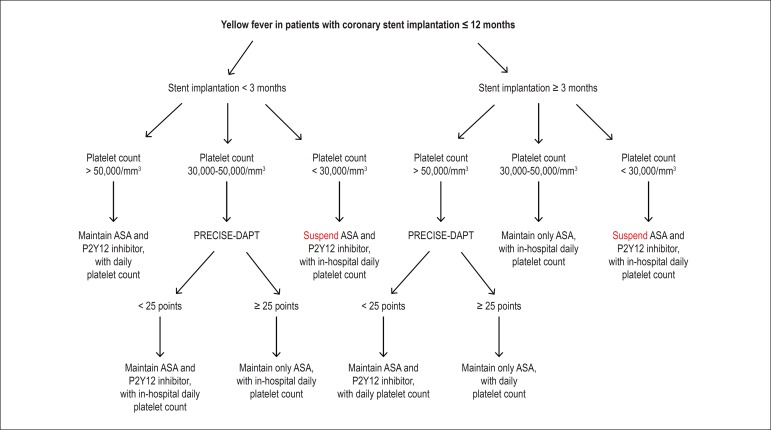
Algorithm for the management of antiplatelet drugs in patients with coronary stents implanted within less than 12 months and yellow fever with neither active bleeding nor blood dyscrasia signs. ASA: acetylsalicylic acid.

The 2017 European Society of Cardiology guideline considers that score in some of its recommendations and raises the possibility of only 1 month of dual antiplatelet therapy for patients at high risk for bleeding (PRECISE-DAPT ≥25), who might not tolerate 3 months of use. Those recommendations and that score application do not depend on the type of stent implanted.^17^ Although the incorporation of that strategy into the management of patients with YF has been neither studied nor validated, it provides additional enhancement to isolated platelet count to estimate the risk for thrombosis and bleeding after percutaneous coronary interventions. Such assessment would be of fundamental importance to define the management, mainly because the modifiable variables used in the PRECISE-DAPT score can be affected by YF. Figure 1 shows an algorithm for the antiplatelet management of patients with coronary stents implanted within less than 12 months from the YF infection.

It is worth noting that in the presence of active bleeding or significant blood dyscrasia secondary to hepatic failure (INR > 1.5 or clotting time > 20 minutes), antiplatelet therapy should be suspended independently of any other criterion. Similarly, the suspension of antiplatelet drugs in patients with CAD without stents, or who had undergone percutaneous coronary interventions more than 12 months before, is recommended, even in moderate cases without significantly low platelet count, because the short-term thrombotic risk of those patients is lower. In addition, oral anticoagulants should be avoided in moderate severity cases, and in-hospital parenteral anticoagulation can be considered for patients with mechanical valve prostheses without active bleeding, evidence of liver dysfunction or other criteria of greater severity.

Patients with heart failure constitute another group whose management might require differentiated approaches in the context of YF. Support therapy in patients with moderate to severe forms of disease depends mainly on the maintenance of an appropriate hemodynamic status through oral or venous hydration, occasional transfusions of blood derivatives and even the use of vasoactive amines. In this scenario, the hemodynamic balance should be constantly reassessed and carefully adjusted, with eventual invasive monitoring in more extreme situations, because those patients are very sensitive to small variations in blood volume.

In addition, the maintenance of drugs often used in the chronic treatment of heart failure, such as diuretics, angiotensin-converting enzyme (ACE) inhibitors and beta-blockers, might hinder the clinical management. Thus, in situations of moderate severity, with neither bleeding nor hemodynamic, renal or respiratory impairment, we suggest maintaining only beta-blockers, preferably at their usual dose. However, they should be avoided in severe cases, with a higher likelihood of clinical deterioration. This recommendation is based on the previous demonstration that the suspension or reduction of those drugs in heart failure proved to be deleterious in other situations of clinical agudization.^18^ Thus, similarly to diuretics and ACE inhibitors, statins should be avoided even in moderate severity cases, mainly because of their potential hepatotoxic effect.

Finally, the vaccine against YF should not be contraindicated based only on the presence of an underlying heart disease, even in patients with previous infarction and/or heart failure. For those patients, the criteria are the same already recommended by the Ministry of Health, with vaccination preferably indicated in the presence of high likelihood of exposure to the virus and low risk for adverse effects.^14^ In the context of heart disease, only transplanted patients should not be vaccinated, because they are on chronic immunosuppressive therapy.

There is an increasing need for further and more detailed studies that assess how arboviral and cardiovascular diseases interact from both the individual and epidemiological viewpoints. In addition, the ineffective control of those epidemics is clearly related to socioeconomic deficiencies and failures in the environmental and urban planning processes, mainly in developing countries. The combination of such factors might be the intersection point, to where investments and research should be primarily directed.
